# The case for paracoccidioidomycosis to be accepted as a neglected tropical (fungal) disease

**DOI:** 10.1371/journal.pntd.0007195

**Published:** 2019-05-16

**Authors:** Joshua Griffiths, Arnaldo Lopes Colombo, David W. Denning

**Affiliations:** 1 The University of Manchester, Manchester, United Kingdom; 2 Division of Infectious Diseases, Universidade Federal de São Paulo, São Paulo, Brazil; 3 National Aspergillosis Centre, Wythenshawe Hospital, Manchester Academic Health Science Centre, Manchester, United Kingdom; 4 Global Action Fund for Fungal Infections, Geneva, Switzerland; Yale University School of Medicine, UNITED STATES

## Introduction

The World Health Organization’s (WHO) neglected tropical disease (NTD) portfolio is a diverse group of diseases with profound impacts on affected populations. The diseases are recognised as being both a symptom of poverty and a powerful contributor to the ‘poverty trap’—a complex and self-perpetuating phenomenon of the interrelated burdens of disease, conflict, poverty, and low educational attainment [1–3]. In 2017, chromoblastomycosis was accepted as an NTD, following the example of mycetoma, by the Strategic and Technical Advisory Group for NTDs (STAG-NTD) and WHO Executive Board with ‘other deep mycoses’ [4].

**Fig 1:**
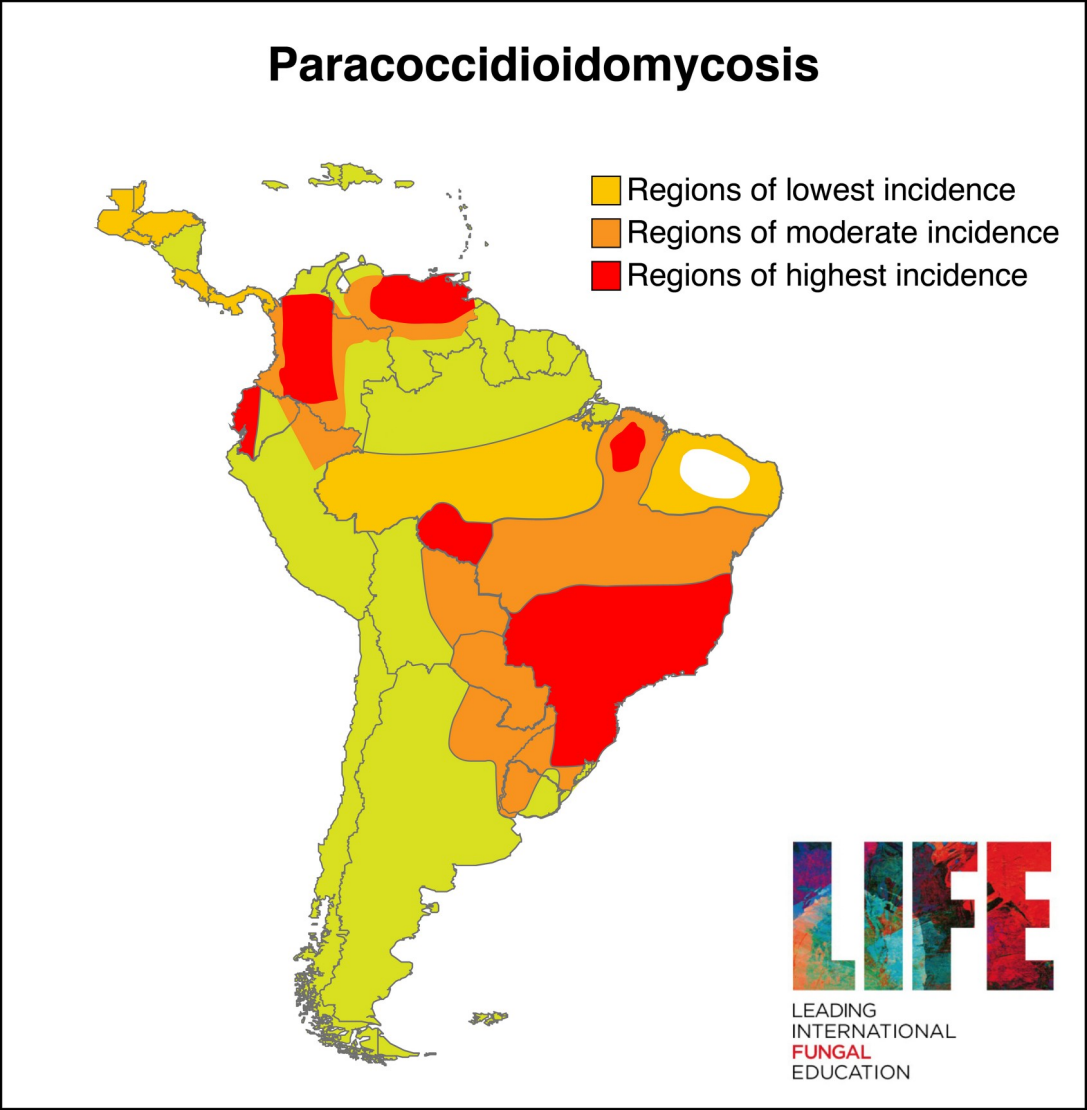
Distribution of paracoccidioidomycosis in South America. Image credit: with permission from Leading International Fungal Education (www.LIFE-worldwide.org).

Paracoccidioidomycosis (PCM) is a deep mycosis endemic to Latin America. Autochthonous cases are exclusive to the tropical and subtropical zone from Mexico (23° north) to Argentina (35° south) (Fig 1), and it is more common in areas with a high rainfall and subject to flooding [5]. It is caused by members of the *Paracoccidioides* genus, a dimorphic fungi found in soil that includes two different species: *Paracoccidioides brasiliensis* and *P*. *lutzii*. It predominantly affects males [6] from rural communities, or those who have had prolonged contact with a rural environment, aged between 30 and 60 years old [7–9].

The impact of this fungus in South America is widespread and devastating. Although already recognised by *PLOS Neglected Tropical Diseases* as a major NTD, we argue here that WHO and the Pan-American Health Organization (PAHO) should explicitly recognise PCM as an NTD.

## Public health impact and association with poverty

Previous reviews of the epidemiology of PCM have attempted to estimate the incidence of the disease using case series [5,10]. Large series published from geographic areas with stable endemicity suggest incidence rates of 1–4 cases/100,000 inhabitants per year in Brazil and Colombia [8,11,12]. In hyperendemic areas from Brazil, annual incidence rates may be as high as 9–40 cases/100,000 inhabitants [10,13]. PCM caused 1,853 deaths in Brazil from 1996 to 2006 [14]. In two recent large epidemiological studies, the mortality of PCM was between 6.1% [13] and 7.6% [15].

Several of the predisposing factors for PCM may be related to poverty. First, as the conidia-producing form of *Paracoccidioide*s spp. resides in soil and host-to-host transmission does not occur, PCM predominately affects those who have had prolonged contact with soil in endemic regions. This explains why the vast majority of cases are seen in patients who have lived or worked rurally—93.5% in a 1,000-case series published in 2011 [8]. It has been described as an occupational disease of farmers [10]. In addition to smoking, high alcohol intake predisposes to the progression of latent foci to active disease [16]. Malnutrition is also thought to be a contributor to the development of the disease [10].

**Fig 2:**
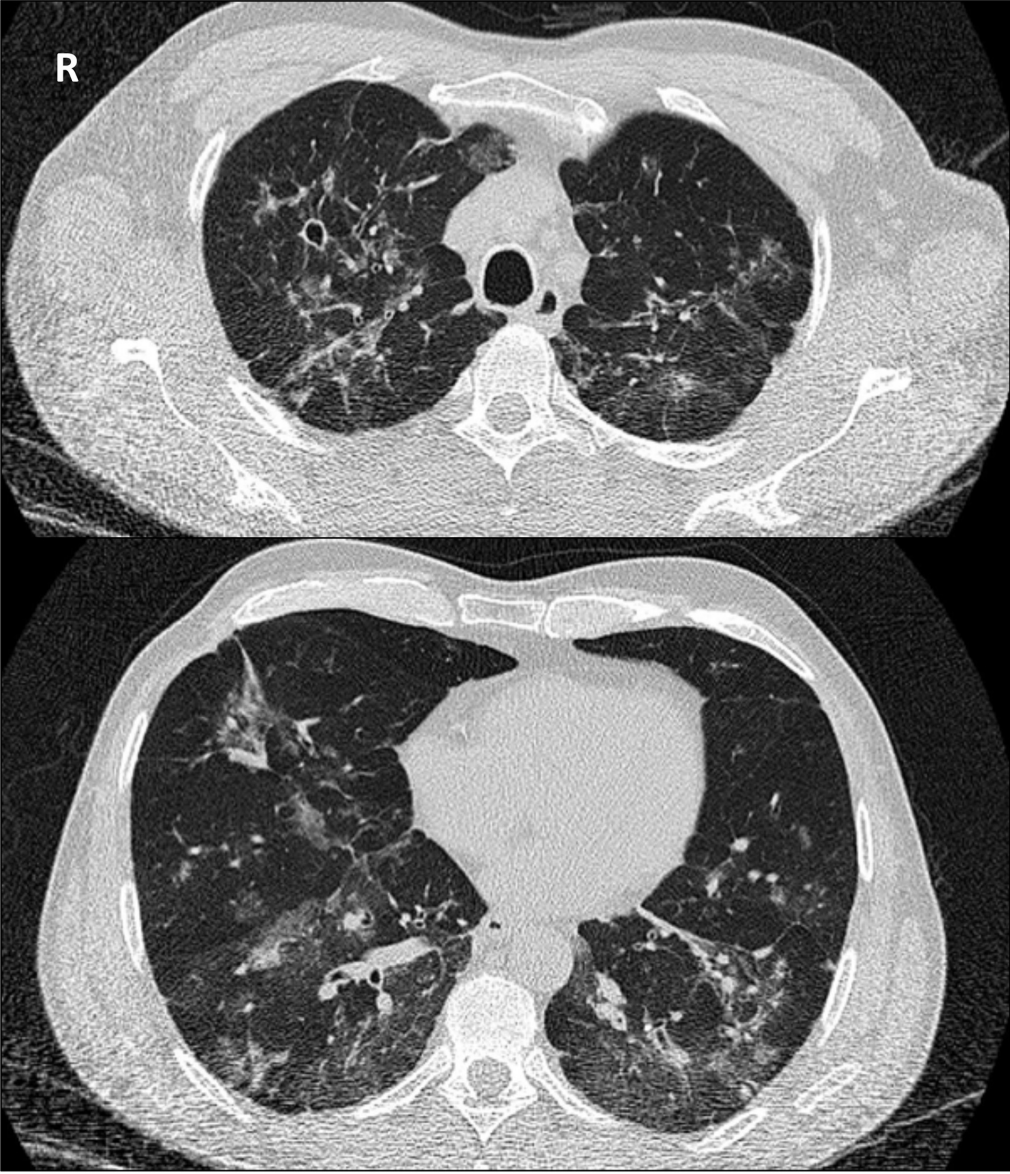
CT scan of pulmonary paracoccidioidomycosis with bilateral small nodules, ground glass changes, and a small cavity in the right upper lobe. CT, computed tomography.

**Fig 3:**
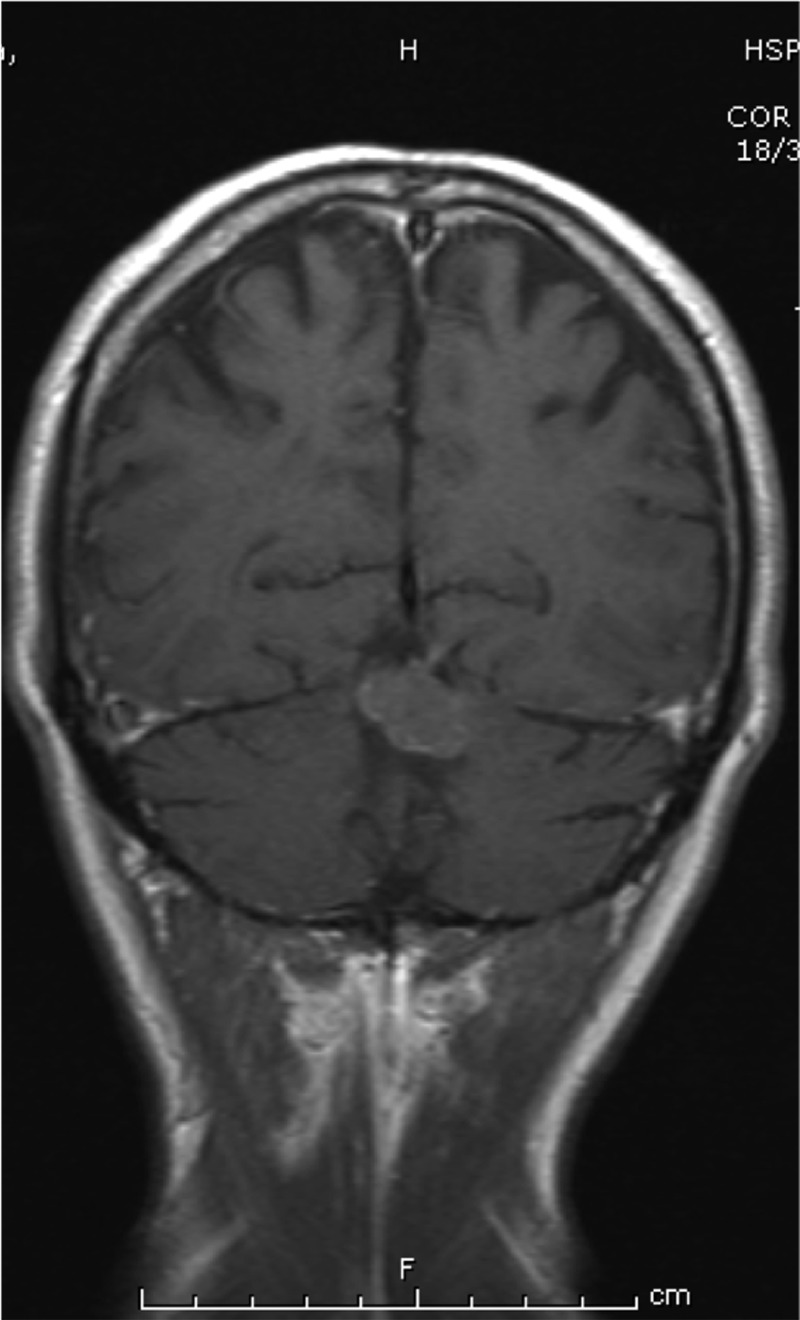
Centrally located neuroparacoccidioidomycosis in the posterior fossa measuring 2.4 × 1.8 cm.

Sequelae in different organs are frequently found as a result of late diagnosis of the fungal infection [15,17]. The sequelae of this disease during a sufferer’s economically most productive period can be significant. Combined with the potentially high cost of prolonged treatment, these factors contribute to this disease’s poverty-inducing potential. The sequelae of this disease are often the result of chronic inflammatory processes, leading to the accumulation of collagen in fibrosis—which may profoundly impact organ function and cannot be treated by antifungal medication [18]. Pulmonary involvement is common and may be confused with tuberculosis, leading to delayed diagnosis (Fig 2). PCM-associated pulmonary fibrosis is irreversible, and its restriction of patients’ activities of daily living can be profound [19]. Lung fibrosis occurs in up to 53% of treated patients with pulmonary PCM [20]. By using high-resolution computed tomography to evaluate 50 consecutive patients with PCM after successful treatment with antifungal drugs, residual radiological abnormalities were found in almost all patients, including architectural distortion (90%), reticulate and septal thickening (88%), centrilobular and paraseptal emphysema (84%), and parenchymal bands (74%) [20]. Adrenal involvement is also very common in PCM. At autopsy, *P*. *brasiliensis* has been demonstrated by direct visualisation of adrenal tissue in up to 90% of patients. In total, 14%–48% of patients with PCM have asymptomatic adrenal dysfunction, demonstrated by limited cortisol response to adrenocorticotropic hormone (ACTH) stimulation, and 3%–7% of patients develop Addison disease [21,22]. Neuroparacoccidioidomycosis (Fig 3) can be particularly disabling, and the risk of sequelae in this form of the disease is high. It may produce motor deficits, epilepsy, or significantly raised intracranial pressure requiring ventral shunting [23]. Dysphonia with vocal cord lesions [24], laryngeal obstruction necessitating tracheostomy, synechia of the buttocks following perianal lesions, and the particularly disfiguring microstomia following facial lesions are among the other sequelae described [25].

Unlike other systemic mycoses such as histoplasmosis and cryptococcosis, an increased incidence of PCM in HIV-infected individuals has not been demonstrated, despite the epidemiological overlap of the two diseases [26]. The clinical course of the disease in patients coinfected with PCM and HIV tends to be more severe. Additionally, patients usually present with pulmonary lesions (a feature of the chronic form, which otherwise is almost the exclusive form affecting those over 30 years old) as well as generalised lymphadenopathy, splenomegaly, bone lesions, and skin lesions as a result of haematogenous dissemination (a feature of the acute/subacute form of the disease, which tends to affect children) [26]. In a 2009 retrospective case control study, the mortality in HIV-positive PCM patients was 12.2% (directly attributable to PCM, 24.4% all-cause mortality) compared with 6% in HIV-negative PCM patients [27]. The relapse rate is also usually higher in HIV-infected patients than that reported in normal hosts, and much longer treatment regimens are usually required.

### Control and Prevention: Ongoing research and development needs

#### Diagnosis

**Fig 4:**
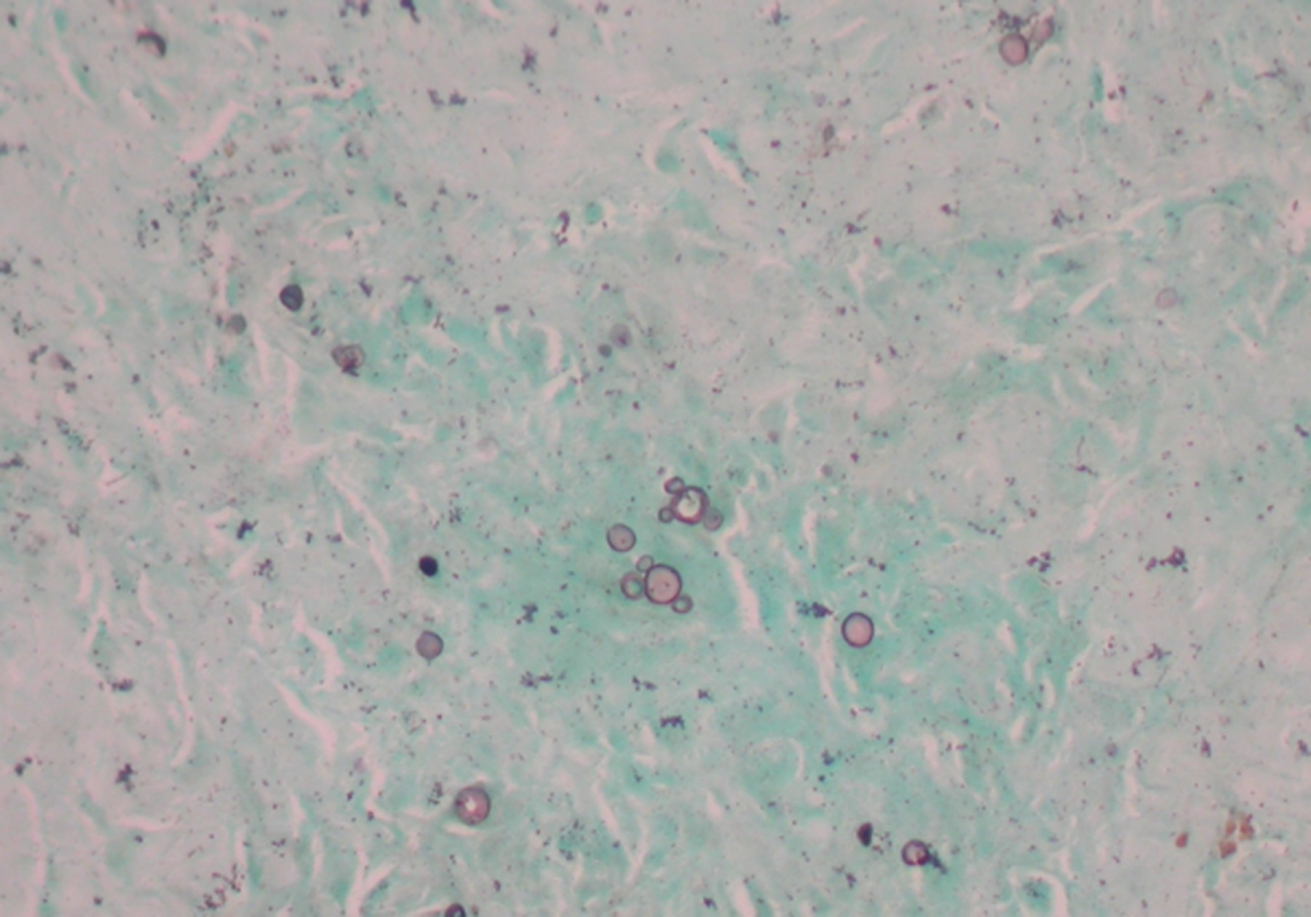
Lung biopsy stained by methenamine silver (Gomori–Grocott) illustrating large yeast cells surrounded by multiple budding. *Photomicrograph provided by Prof Rimarcs Gomes Ferreira*, *Department of Pathology-Escola Paulista de Medicina-UNIFESP*. UNIFESP, Universidade Federal de São Paulo.

The ‘gold standard’ for the diagnosis of PCM is direct visualisation of the fungal yeast cells surrounded by multiple budding daughter cells (the ‘pilot’s wheel’) or isolation of the fungal agent in culture of clinical samples or tissue (Fig 4) [28]. The difficulties of demonstrating the fungus in clinical samples and the length of time that confirmation by culture requires means that serological tests have vital application in both the diagnosis of PCM and in monitoring treatment response (Table 1) [29]. Double immunodiffusion (DID) is the serological test of choice for the diagnosis of PCM. There are some key current problems in the serological diagnosis of PCM. The first is that the new insights into the complexity of the *Paracoccidioides* genus are shedding light on the wide variation in antigen production. For example, the antigenic 43-kDa glycoprotein gp-43 is the main antigen used in this test in the serological diagnosis of PCM, but some isolates in the *Paracoccidioides* spp. complex produce either very low levels of or no gp-43 antigens—particularly *P*. *lutzii* [29]. gp-43 is therefore not recommended as a single-antigen preparation for diagnosis. Furthermore, preparation of the antigens for serological diagnosis is not standardised with variations in production, resulting in substantial variation in immunogenicity. Mixtures of unidentified antigens are often used, which may impair the tests’ sensitivity and specificity because of cross-reactivity with the sera from patients with other mycoses [29].

**Table 1:** Diagnostic methods for PCM and evaluation of value and drawbacks.

Diagnostic methods	Sensitivity	Specificity	Pros	Cons
Serology (DID, CIE, IIF)	69%–100%	80%–100%	• Correlates with the severity of disease • Monitor therapeutic response • Inexpensive	• No commercial kits available • No standardization, impairing reproducibility and repetitiveness No validated serological techniques for *P*. *lutzii* • May be negative in immunosuppressive conditions • Cross-reaction with histoplasmosis and aspergillosis
Specific antigen detection gp43kDa and gp70kDa	100%	96%	• Provides diagnosis in immunocompromised patients with negative production of specific antibodies • Provides diagnosis in biological materials with low burden of infection by detecting specific fungal antigens (serum, BAL, CSF)	• Expensive when compared with conventional methods • Not available for routine diagnosis of PCM
Fresh examination/ direct microscopy	48%–75%, worst in sputum	HIGH	• Immediate results • Samples are easy to obtain • Inexpensive	• Requires skilled professionals to read the exam Micromorphology of *P*. *brasiliensis/P*. *lutzii* pathogens are not distinguished
Culture	25%–44%	100%	• Provides material for further evaluation of species, antifungal susceptibility, and virulence	• 2–6 weeks of incubation • Biohazard concerns
Histopathology	65%–97%	HIGH	• May help to define the severity of disease (compact granuloma versus loose granuloma) • Requires skilled professionals	• Invasive procedure is required for biopsy Small forms of *Paracoccidioides* spp. might be confounded with *Histoplasma capsulatum* or *Cryptococcus neoformans*
Molecular methods (PCR)	HIGH	HIGH	• Provides species identification • Provides diagnosis in biological materials with low burden of infection	• Expensive when compared with conventional methods • Not available for routine diagnosis of PCM

Abbreviations: BAL, bronchoalveolar lavage; CIE, counterimmune electrophoresis; CSF, cerebrospinal fluid; DID, double immunodiffusion; gp43kDa, gp43kDa gene of *P*. *brasiliensis*; gp70kDa, gp70kDa gene of *P*. *brasiliensis*; IIF, indirect immunofluorescence test; PCM, paracoccidioidomycosis.

These two factors combined help explain the large interlaboratory variations in diagnosis recently demonstrated. In 2014, Vidal and colleagues compared serological diagnosis between six major Brazilian reference centres. It was found that there was a high rate of major discordance (20%), which can affect clinical decision-making. Those centres using antigens from pooled isolates performed better [30].

New PCM serum markers and a standardization of the diagnostic approach are required in order to effectively diagnose PCM and thereby manage it at a public health level. Past attempts at standardisation have foundered, as reference centres have used ‘in-house’ methodologies for many years, often with little feedback from clinicians, and there is little impetus for change [30]. Using standardised, purified, cloned antigens may be a way forward, rather than standardising ‘in-house’ antigen production. Using purified antigens reduces the risk of cross-reactivity, thereby increasing specificity, and their use, particularly in combination, can lead to high levels of sensitivity and specificity [29]. Otherwise, ‘point-of-care’ tests would certainly be more suitable for providing early diagnosis in endemic regions.

Education at all levels of the healthcare system is also required, and a free online course on microscopy and histology has recently been launched by Leading Fungal Education International in four languages (www.microfungi.net).

### Treatment

There is a lack of a high-quality body of evidence guiding the treatment of PCM. It is responsive to many antifungal medications. In mild to moderate disease, a 200-mg dose of itraconazole was recommended as the first-line choice in the 2017 Brazilian consensus guidelines [31]. In severe PCM, the use of intravenous amphotericin B preparations, either as deoxycholate or in a lipid formulation, or alternatively intravenous cotrimoxazole was recommended, although there are very few data published [31]. Only two randomised trials of treatment regimens for PCM have been conducted, one comparing itraconazole and voriconazole [31] and the other comparing itraconazole, ketoconazole, and sulfadiazine [32]. Neither had the statistical power to demonstrate superiority of one treatment over the other. The most recent guidelines are based on numerous noncomparative studies and expert opinions and two studies comparing itraconazole and cotrimoxazole.

The first dual-cohort, nonrandomised study compared itraconazole (200 mg once daily) and cotrimoxazole (1,440 mg every 12 hours) in 177 patients in the induction and maintenance phases of treatment. Although no difference was found in efficacy and effectiveness of reaching either clinical cure in the induction phase or serological cure in the maintenance phase, itraconozole induced cure more quickly in both phases (105 days versus 159 days to clinical cure [*p* = 0.001], 161 days versus 495 days [*p* = 0.02]). It was also associated with fewer side effects (6.4% versus 20% [*p* = 0.03]) [33]. Another comparative cohort study of 200 patients was performed comparing once-daily itraconazole and twice-daily cotrimoxazole [34]. Itraconazole was significantly superior, and the time to cure was 11 months shorter with itraconazole (12 months versus 23 months). Adherence to treatment could be one factor responsible for better outcomes [34].

Generic itraconazole is only 1.6 times more expensive than cotrimoxazole in Brazil when this shortened treatment regime is taken into account. Given a better side effect profile and probable superiority, it is recommended as a first-line therapy [33]. However, sulfamethoxazole-trimethoprim is currently distributed free of charge by the Brazilian Ministry of Health [35] and, as such, is much more commonly used. This anomaly should be modernised.

There are other research areas requiring considerable development and funding that have the potential to benefit from a WHO NTD disease status. Prolonged treatment is currently required for the recovery of cell-mediated immunity. There has been some promising work on the development of a therapeutic vaccine that would induce immunity in experimental models and *in vitro*, which might eventually allow significantly shorter treatment regimes [36]. There is also some evidence in the literature that PCM patients with severe inflammation may benefit from adjunctive therapy with corticosteroids [37].

### Epidemiology and prevention

The 2017 Brazilian guidelines for the management of PCM called for the development of a national registry of cases and the institution of compulsory notification [31]. Such a registry would be beneficial in many ways, giving a more accurate understanding of the size and spread of the problem and allowing more effective strategic healthcare provision planning and commensurate allocation of resources.

Prevention of PCM is a challenge given the very high rates of infection and the apparent ease of exposure to the fungus in rural areas. Current recommendations advise avoiding exposure to soil dust in endemic areas if possible (particularly in children and the immunocompromised) and the use of N95 respirators or well-sealed cabins in agricultural machinery for rural workers otherwise unable to avoid exposure in hyperendemic areas. Health education programmes, particularly in hyperendemic areas, could tackle both exposure to the fungus and the usual delayed presentation of the chronic disease to medical attention. These programmes could also be extended to urban centres receiving high volumes of migrants from these areas. No efforts to treat latent PCM, as for tuberculosis, are published. Much more work is needed to develop such effective public health interventions and assess their value.

## Conclusion

PCM fulfils WHO NTD criteria and would benefit from such a classification. PCM is endemic to the tropical regions of Latin America. PCM is an important cause of mortality in Brazil [14,38]. PCM causes significant morbidity and predominately affects poor rural workers or people living in urban slums at their most economically productive stage of life and requires very long treatment regimes. Lung sequelae are frequent, reducing work productivity and quality of life of a large number of patients, and other complications can also be very disabling.

PCM is neglected by research, and the care of patients with the disease is underdeveloped when it comes to new diagnostics, medicine, and other control tools. There are currently significant problems in the diagnosis of PCM, with a great need to standardise diagnostics and find new serum markers, as well as to develop useful point-of-care tests. In addition, more research is required to optimise treatment—particularly through randomised control trials—and to address latency. Though itraconazole is the first-line recommended drug, it is currently not provided free of charge in most medical centres in Brazil and Latin America. The creation of a national registry of the cases of the disease across all states in Brazil and in other countries in which the fungus presents a public health problem would greatly aid the public health effort.
